# Nanotechnology-Based Bioactive Antifeedant for Plant Protection

**DOI:** 10.3390/nano12040630

**Published:** 2022-02-14

**Authors:** Melanie Melanie, Mia Miranti, Hikmat Kasmara, Desak Made Malini, Teguh Husodo, Camellia Panatarani, I Made Joni, Wawan Hermawan

**Affiliations:** 1 Department of Biology, Faculty of Mathematics and Natural Sciences, Universitas Padjadjaran, Jln Raya Bandung-Sumedang KM 21, Sumedang 45363, West Java, Indonesia; melanie@unpad.ac.id (M.M.); mia.miranti.rustama@unpad.ac.id (M.M.); hikmat@unpad.ac.id (H.K.); desak.made@unpad.ac.id (D.M.M.); t.husodo@unpad.ac.id (T.H.); 2 Functional Nano Powder University Center of Excellence, Universitas Padjadjaran, Jln Raya Bandung Sumedang KM 21, Sumedang 45363, West Java, Indonesia; c.panatarani@phys.unpad.ac.id (C.P.); imadejoni@phys.unpad.ac.id (I.M.J.); 3 Department of Physic, Faculty of Mathematics and Natural Sciences, Universitas Padjadjaran, Jln Raya Ban-dung-Sumedang KM 21, Sumedang 45363, West Java, Indonesia

**Keywords:** biopesticides, antifeedant, nano-delivery system, nanotechnology, plant protection, pest resistance

## Abstract

The productivity of vegetable crops is constrained by insect pests. The search for alternative insect pest control is becoming increasingly important and is including the use of plant-derived pesticides. Plant-derived pesticides are reported as effective in controlling various insect pests through natural mechanisms, with biodegradable organic materials, diverse bioactivity, and low toxicity to non-target organisms. An antifeedant approach for insect control in crop management has been comprehensively studied by many researchers, though it has only been restricted to plant-based compounds and to the laboratory level at least. Nano-delivery formulations of biopesticides offer a wide variety of benefits, including increased effectiveness and efficiency (well-dispersion, wettability, and target delivery) with the improved properties of the antifeedant. This review paper evaluates the role of the nano-delivery system in antifeedant obtained from various plant extracts. The evaluation includes the research progress of antifeedant-based nano-delivery systems and the bioactivity performances of different types of nano-carrier formulations against various insect pests. An antifeedant nano-delivery system can increase their bioactivities, such as increasing sublethal bioactivity or reducing toxicity levels in both crude extracts/essential oils (EOs) and pure compounds. However, the plant-based antifeedant requires nanotechnological development to improve the nano-delivery systems regarding properties related to the bioactive functionality and the target site of insect pests. It is highlighted that the formulation of plant extracts creates a forthcoming insight for a field-scale application of this nano-delivery antifeedant due to the possible economic production process.

## 1. Introduction

Global use of pesticides has continued to grow over the last few decades, with explosive growth especially in Asia and America [1]. Although the application of pesticides has shown a real impact on environmental and human health problems, it seems that farmers depend on synthetic insecticides to combat insect pests [2]. Thus, high dependency on chemicals has caused increasing insect pest resistance and high-cost crop protection [3]. Moreover, the impact of resistance triggers insect pest outbreaks that cause massive crop losses [4]. Efforts to overcome these issues have had a real impact on environmental pollution prevention due to the reduced presence of residue in food and agricultural commodities. Biopesticides are listed as eco-friendly pest control agents obtained from living organisms or natural products [5]. Biopesticides that are obtained from natural products as secondary metabolite compounds deriving from plants include alkaloids, terpenoids, phenolic, and other chemical compounds [5].

Generally, synthetic pesticides exert their effect on the nervous system of insects with acute lethal effects and can trigger insect pest resistance [6]. Contrarily, bioinsecticides with systemic bioactivity and indirect toxicity to insects can anticipate the occurrence of insect pest resistance. Therefore, bioinsecticides possess the properties of antifeedant compounds caused by deterrent insect feeding activity, repellents, attractants, developmental inhibitors, and ovipositor deterrents, which are representative of eco-friendly plant protection [7]. The advantages of antifeedants are that they are less toxic than synthetic pesticides with more specific bioactivity against certain insect pests [4,5,8].

Despite many advantages in terms of bioactivity characteristics, bioinsecticides under a field environment easily degrade and the volatile nature of their active compounds leads to inefficiency in field applications [9]. Thus, these characteristics are responsible for reducing their efficacy. To resolve those challenges, many researchers are interested in the development of efficient bioinsecticide formulations that allow effective agricultural management [9,10,11]. In this regard, the use of nanotechnological tools to resolve inefficient bioinsecticide formulations is very important for the development of beneficial and prospective biopesticide formulas [5,7,12,13].

**Figure 1:**
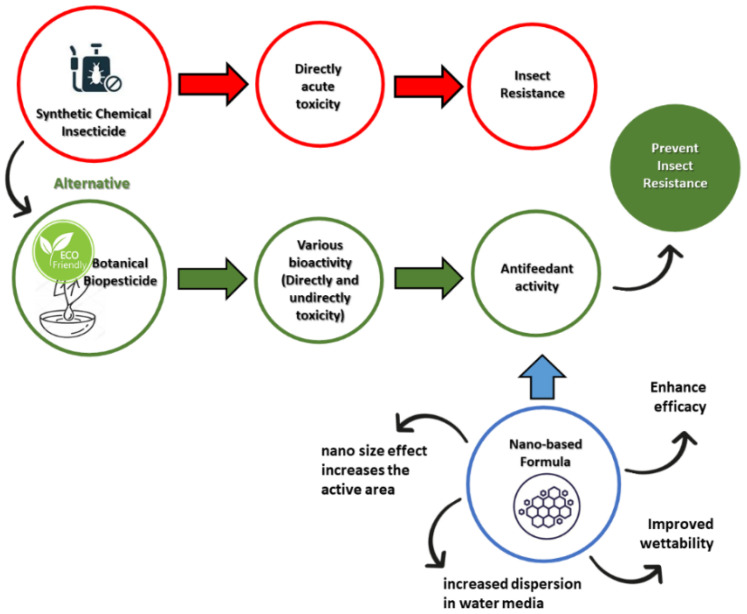
The negative impact of synthetic insecticides and effort of developing nanoformula-based antifeedant as an alternative to prevent pest resistance.

A nano-based formulation aims to improve the properties of biopesticides [10,14]. The conventional formulation is initially developed based on simple solvent-based solutions of emulsifiable concentrates (ECs) [15,16] or a powder mix including the formula of wettable powders (WPs) [17,18]. New generation formulations can be developed through oil-in-water (EW) emulsion [19,20] and the microemulsions (MEs) formula to achieve homogeneous and isotropic dispersions of the formula [21,22]. The most recent nanoemulsions (NEs) formula involves fine oil-in-water dispersions with a droplet nano-size (1–100 nm) prepared using an appropriate surfactant that is soluble in organic compounds and water and, consequently, provides kinetic stability [23,24]. Furthermore, de Oliveira et al. [12] outline the challenges for the nano-scale formulation of botanical pesticides. The highlight of the study is the benefits of the formula’s nano-based properties to improve the efficacy, solubility, dispersity, and also wettability of the active ingredient in the water-based system [12]. Additionally emphasized is the use of organic materials as a potential carrier system supported by the active ingredients. Despite many developments on bioinsecticide with a nanoformulation, and also its extensive application, environmental issues such as pest resistance still occur. Thus, an alternative solution, such as antifeedant along with its nano-based formulation, in comparison with conventional synthetic insecticide is illustrated in Figure 1. The advantages of a nanotechnology-based formulation for antifeedant provide an alternative to prevent the negative impact of synthetic insecticides that may cause pest resistance.

Therefore, the study of insect antifeedant on crop protectants has attracted many researchers [7,25,26,27,28,29]. The active compounds of antifeedant do not kill pests directly but they cause starvation or predation by their natural enemies. Thus, the affectivity of antifeedant deployment in the field with various environmental conditions requires more creative strategies [25]. A recent important review reports on antifeedant strategies involving the role of nanotechnology approaches and opportunities. Koul [29] and Isman [7] evaluated that the majority of antifeedant studies still rely on an investigation of the antifeedant’s source as being from a variety of essential oils and crude plant extracts including their bioassays due to the laboratory scale of plant extract screening. Hence, a nano-based formulation to achieve desirable properties is required in antifeedant application strategies.

The characteristics and properties of the antifeedant delivery system govern the feeding activity of phytophagous insects quantified by their insect feeding trace (consumed leaf area). Referring to a study focused on biopesticide nano-delivery formula, the pesticide loaded in colloidal or the encapsulation of botanical pesticides in nano-structured systems has emerged as an important tool to improve the quality of the formulations [11,30,31,32,33,34]. However, botanical pesticides are usually soluble in an organic solvent, thus making a formula in water solvent requires additional ingredients to facilitate suitable delivery formulation. Aqueous nanoemulsion, nanosuspension-loaded essential oil, or unsolvable solid organic compounds in micelle can increase the solubility and dispersion in aqueous solution, improving the wettability, efficiency, and stability that can enhance the bioavailability properties during application [9,32].

Generally, nanoformulation and nanoencapsulation delivery formulas aim to enhance efficacy and maintain the durability of the active ingredients through a slow-release mechanism on the targets [35,36]. There are a large number of studies revealing nano-delivery formulas of plant biopesticides [11,31,37,38]. Recently, many studies have reported efforts on the formulation of a nano-carrier of essential oils and plant extracts for antifeedant-targeted delivery against insect pests [39,40]. However, there are no review studies related to antifeedant nano-delivery-based formulas, especially regarding the active compounds obtained from plant extracts. This review discusses several recent studies focusing on pest control strategies using botanical pesticides developed in nano-delivery formulas. In particular, it discusses the improvement in properties for the effectiveness and efficiency of antifeedants in controlling insect pests. Thus, this study provides a strategy in pest control to overcome insect resistance due to the dependence on using synthetic insecticides. The review is a scoping review, which was compiled using references (last 10 years) from major databases such as Science Direct, PubMed, MDPI, ACS Database, SpringerLink, Google Scholar, Taylor Francis, and Open Knowledge Maps based on scoping areas of research in the field of nano-biopesticides delivery and antifeedant-based nanoformulation.

## 2. The Opportunity of Antifeedant Nano-Based Delivery Systems

### 2.1. Insect Pest Control Strategies Using Antifeedant Plant-Derived Pesticides: Antifeedant Management, Resources, and Reserve

The concept of pest control today has developed on the principles of integrated pest control that considers economic threshold aspects, application of biopesticides from natural products, and biocontrol using natural enemies or agencies entomopathogenic [4,41,42]. Certainly, Integrated Pest Management (IPM) is a combination of the conventional approach and the adoption of various technological developments, which plays an important role in achieving sustainable agriculture goals [43]. Bioinsecticides are part of the strategies that significantly contribute to IPM [4]. The multi-active role of plant-based bioinsecticides has been studied, which has included toxicity and growth inhibitory effects, and the role of antifeedant on preventing insect resistance [29,44]. Moreover, the environmentally friendly nature of preventive approaches such as antifeedant makes them good alternatives for insect pest control strategies.

An antifeedant received from the secondary metabolite active compounds of plants revealed phytophagous insect protection [4,45]. These active compounds belong to a group of allomones, which are interspecific compounds needed to mediate the interaction with phytophagous insects [45]. The groups of metabolite active antifeedant compounds that have been reported are limonoids, chromenes, polyacetylenes, saponins, flavonoids, quassinoids, cucurbitacins, cyclopropane acids, phenolics, alkaloids, various types of terpenes, monoterpenoids, diterpenoids, sesquiterpenes, and their derivatives [46,47,48]. There are two fundamental mechanisms of antifeedant; the first being the deterrent effect due to taste receptors, and the second being due to disruption of the midgut of the insects. The deterrent effect is due to taste receptors being stimulated by the phagostimulant compound interfering with the normal function of neurons [26,29,46]. Meanwhile, Isman [25] has stated that deterrent effect activities caused by chemicals serve to accelerate the roles of the central nervous system in preventing ingestion or absorption of substances and, subsequently, creating sublethal toxicity and disruption of the midgut.

Studies over the past 10 years on bioinsecticide antifeedant, including the reserves and preparation, the bioactive compounds, the pest control, and the insect pest targets, are summarized in Table 1. The investigations of potential antifeedants have been extensively studied in the field of crop protection. The order Lepidoptera dominates as the main insect pest on crops due to its shorter life cycle characteristic and higher fecundity capacity, among others [49]. In addition, antifeedants explore stored grain insect pest control and play a small part in oil palm plantation insect pest management. Meanwhile, the majority of grain-stored insect pests is dominated by the order Coleopteran [50], where their feeding activities on crushing grains into powder caused them to be easily contaminated by fungi and bacteria. In addition, carcasses, secretions, eggs, and fecal remains cause allergic reactions in humans. To overcome this problem, the use of synthetic insecticides and fumigation turned out to leave residues on grains that are harmful to consumers. However, these insect pests tend to be adaptable and more resistant to synthetic chemical insecticides by inheriting resistant generations. As an alternative, bioinsecticide antifeedant provides a variety of compositions of phytochemical compounds, allowing various modes of action to prevent such resistance problems from occurring [4]. Therefore, this encourages researchers to use antifeedants as prospective alternatives for crop protection and stored pest management.

Regarding plant-source bioactivity on stored grain insect pests, semiochemical repellents provide excellent performance compared to antifeedants. Thus, semiochemical repellents are more rapidly developed in stored pest control strategies [51]. On the other hand, the use of antifeedants to control oil palm pests is driven by the importance of maintaining natural enemies to prevent a resurgence due to the irresponsible overuse of synthetic insecticides [52]. The damage of oil palm plantations is due to the activity of the polyphagous insect pest that defoliates young palm plantations. Although antifeedants play an important role in controlling feeding activity and maintaining the existence of natural enemies, their role in oil palm pest control strategies seems unexplored at an intensive level.

**Table 1:** The antifeedant strategies.

No.	Pest Control Strategies	Resources	Reserve/Formulations	Preparation Methods	Bioactive Compounds	Efficacy	Target Insect Pests	Ref.
1	Stored grain pest manage-ment	Sunflower seed (*Helianthus annuus*)	Crude oil of sunflower seed hulls (SSH)	Pyrolysis reactor used to produce bio-oils	Acetic acid, furfural, methoxyphenol	*Higher antifeedant * FDI (79.3%) ECI (87.5%) *Moderate antifeedant * FDI (45.8%) ECI (16.6%)	*Sitophilus oryzae*, *Lasioderma serricorne* and *Tribolium castaneum*	[53]
		*Mentha piperita*	Essential oil	Hydrodistillation	Menthone and menthol	*Antifeedant activity* *S. oryzae* FDI (29.68%, 18.81%, and 14.34%) *T. castaneum* FDI (20.67%, 13.73%, and 9.19%)	*Sitophilus oryzae* Linn. and *T. castaneum*	[54]
		*Gaultheria procumbens*	Essential oil	Hydrodistillation	Methyl salicylate (MS), citral, Z-citral	*Antifeedant activity* FDI 8.26% (EO) FDI 5.33% (MS)	*Sitophilus oryzae* and *Rhyzopertha Dominica*	[55]
		*Zanthoxylum bungeanum*, *Z. schinifolium*, *Z. armatum* and *Z. dissitum*	Methanolic stem bark extract	Screening active fraction continued by active compound isolation	Benzophenan-thridines	*Antifeedant activity* FDI (41.12%) EC_50_ 62.67 ppm (norche chelerythrine) EC_50_ 66.97 ppm (decarine)	*Tribolium castaneum*	[56]
2	Crop protection management	*Melia volkensii*	Methanolic crude extracts from the bark, leaves, pulp, and nuts	Dry crude extracts dissolved in methanol and topped up with water (96% of total volume) with several concentrations used for in vivo and in vitro assay	1α,3α-Diacetylvilasinin, 1-cinnamoyl-trichilinin, 1-tigloyltrichilinin, 1-acetyltrichilinin, salannin, 1-detigloyl-1-isobutyl-salannin, 20,30-dihydro-salannin, ohchinin3-acetate, nimbolin B, volkensin, and toosen-danin	*Antifeedant activity* *C. Puncticollis.* FDI (51%) (nut and pulp) FDI (43%) (bark) FDI (44%) (leaf) *S. exigua*. FDI (57%) (nut and pulp) FDI (58%) (bark) FDI (50%) (leaf)	*Cylas puncticollis*, *Spodoptera exigua*, *S. Frugiperda*	[57]
		*Lantana Camara*	Ethyl acetate active fraction (EAF) leaves *L. camara*	Nanosuspension formulation by reverse emulsion with the various components of Tween 80 as a surfactant	Alkaloids, saponins, and steroids	*Strong antifeedant activity* SOR 11 at the LC_50_ 0.39% (D = 8.3 ± 1.3 nm), leading to wettability improvement on the leaf surface	*Crocidolomia pavonana* Fab.	[58]
		*Annona mucosa* Jacq.	Ethanolic seed extract *A. mucosa* and biopesticide of *A. squamosa* extraction (Anosom^®^ 1 EC)	Emulsifiable concentrate formulation of ESAM	Rolliniastatin-1 (ESAM), fatty acid-derived substances contain acetoge-nins (Anosom^®^ 1 EC)	*Strong antifeedant activity* Decreased foliar consumption (>90%) at LC_50_ = 411.55 and 312.08 mg kg^−1^ in 168 h (crude extract and ESAM)	*Helicoverpa armigera*	[59]
		*Panax ginseng*	Methanolic stems and leaves extract	Isolated pure compounds from the methanolic crude extract determined using the HPLC method	Ginsenoside	*Moderate antifeedant activities* (AFC_50_) are 4.98 and 5.03 mg mL^−1^ at 24 h and 48 h (no-choice assay), and 2.74 and 4.14 mg mL^−1^ (choice assay) (the pure compounds)	*Plutella xylostella*	[60]
		*Pilgerodendron uviferum*	Essential oil (EO), petroleum ether extract (PEE), and dichloromethane extract (DCME)	Fractioned EO and both of the extracts by column chromatography with silica gel as a stationary phase, using a different gradient elution for each extract	Sesquiterpenes (circa 60%): (−)-trans-calamenene, cadalene, (−)-cubenol, (−)-epicubenol, (−)-torreyol, (−)-15-copaenol	*Antifeedant activities* (EO, DCME, and the sesquiterpenes isolated pure compounds)	*Hylastinus obscurus*	[61]
		*Cyperus eragrosits*	Nature and synthetic compounds of dihydro benzofurans and aurones	Organic synthesis and electrochemical oxidation	Acetophenone-type dihydro benzofurans, coumaran (aurone derivatives)	*Higher antifeedant activity* of natural aurones compared with synthetic compounds	*Spodoptera litura* and *Plutella xylostella*	[62]
		*Caesalpinia bonduc* (L.) Roxb.	Chloroform extract	Screening crude extracts and fractions solvents with increasing polarity	Coumarins, flavonoids, terpenoids, phenols, and quinones	*Higher antifeedant* of fraction 3 of chloroform EC_50_ = 357.13 ppm than the crude chloroform extract that shows a minimum EC_50_ 3.07%	*Helicoverpa armigera*	[63]
		*Millettia pachycarpa*	Hexane and methanol leaves extracts	Screening active fraction	Flavonoids and isoflavonoids (mille-wanins, prenylated isoflavones, and pyra-nochal-cones)	*Strong antifeedant activity* AI_50_ = 227.12 ppm at 24 h (the hexane) *Good antifeedant activity* AI_50_ = 340.87 ppm at 24 h (the methanol extract)	*Spodoptera litura*	[64]
		*Azadirachta indica*	Crude neem gum from the incised bark of *A. indica*	Neem gum nano formulation (NGNF)	Hexadecanoic acid, oleic acid, and ricinoleic acid	*Strong antifeedant activity* FDI (100%) at LC_50_ 10.20 ppm (NGNF)	*Helicoverpa armigera* and *Spodoptera litura*	[40]
		*Pinus kesiya* Royle., *Lantana Camara* Linn., *Litsea cubeba* Lour., *Gaultheria fragrantissima* Wall., *Mikania micrantha* Kunth., *Ambrosia artemisiifolia* Linn., and *Eupatorium riparium* Regel., the indigenous plants of Meghalaya	The methanolic crude extracts of leaves and aerial parts of plants	Extraction by Soxhlet method	Alkaloids, flavonoids phenols, phytosterols, saponins, tannins, and terpenoids	*Higher antifeedant activity* FDI (50.92%, 70.61%) at 0.1% and 0.5% concentrations extract of *G. Fragrantissima* than extract of *L. cubeba* *Moderate antifeedant activity* Extract of *P. kesiya*	*Helicoverpa armigera*	[65]
		*Cabralea canjerana* canjerana	Fruits and seeds extracts	Fractionation (ethyl acetate and ethanolic fractionation)	Dammarane triterpene, ocotillone 7,15 diol	*Higher antifeedant activity* of crude extract and ethyl acetate seed active fraction compared to pure compounds	*Spodoptera frugiperda*	[66]
		*Acalypha fruticosa* Forssk.	Leaves extract	Dichloromethane extraction	Triterpenoids, steroids, tannins, saponins, flavonoid alkaloids	*Significant antifeedant activity* of the dichloro-methane extract against *L. orbonalis* (77.1%), *H. armigera* (66.2%), *S. litura* (74.8%), and *E. Vittella* (67.2%) followed by acetone, dimethyl sulfoxide, and aqueous extracts	*Leucinodes orbonalis*, *Helicoverpa armigera*, *Spodoptera litura* and *Earias vittella*	[67]
			Hexane, chloroform, and ethyl acetate leaves extracts	Screening active fraction	Terpenoids, tannins, coumarins anthraquinones, and saponins	*Antifeedant activity* FDI (92.8%) at 5% concentration, LC_50_ 1.86% (the cloroform extract) FDI (84.3%) at 1000 ppm concentration, LC_50_ 385.7 ppm (the active fraction)	*Plutella xylostella*	[68]
		*Soymida febrifuga*	Methanolic leaves extract	Isolated pure limonoids compounds determined using H-NMR spectra analysis	Limonoids (phragmalin-type)	*Antifeedant activity* AI (76.46 µg cm^−2^, 66.61 µg cm^−2^) against *A. Janata,* the pure isolated compounds of fissinolide AI (61.69 µg cm^−2^, 51.93 µg cm^−2^) against *S. litura* (Swietenitin)	*Spodoptera litura* and *Achaea janata*	[69]
		*Foeniculum vulgare*, *Anethum graveolens*, *Petroselinum crispum*, *Cuminum cyminum*	Essential oil	Hydrodistillation	Estragole, fenchon, trans-anetholes, carvone, myristicin, cumin aldehyde	*Antifeedant activity* FDI (99.7%) (EO *P. crispum*) FDI (84.7%) (EO *A. graveolens* fruits) FDI (92.4%) (EO trans-anethole) FDI (84.7%) (EO cumin aldehyde)	*Pseudaletia unipuncta*	[70]
		*Syzygium aromaticum* L., *Cinnamomum zeylanicum* Blume, *Lavendula latifolia* L., *L. angustifolia* L., *Mentha crispa* L., *M. arvensis* L. and *M. piperita* L.	Essential oil	Screening EO and pure compounds (single mixture and active compound)	Major constituents: eugenol, (E)-cinna-maldehyde, linalool, n-carvone, menthone, menthol Minor constituents: isoeugenol, β-caryo-phyllene, ceraniol	*Higher antifeedant activity* (DC_50_ = 12.5 and 16.4 μg cm^−2^) *C*. *zeylanicum* and *S. aromaticum* EO are better than pure compounds (eugenol, isoeugenol, and mixture minor compounds)	*Trichoplusia ni* Hubner	[71]
3	Oil palm plantation pest management	*Syzygium aromaticum*	Essential oil-based eugenol compounds	Isolated pure eugenol compounds from clove oil extract	4-Allyl-2-methoxy-1-(4-tri-fluoromethyl-benzyloxy)- benzene	*Highest antifeedant activity * FDI (64.42%) of the pure compounds	*Rhyncho-phorus ferrugineus*	[72]
		*Cymbopogon nardus* and *C. martinii* grown in Colombia	Essential oil	Hydrodistillation	Geraniol	*The higher antifeedant* activity of EO (*C. nardus* and *C. martinii*) is better than pure compound (geraniol) and synthetic repellent IR_3535_	*Euprosterna elaeasa* and *Acharia fusca*	[73]

Note: FDI = Feeding Deterrence Index ECI = Efficiency of Conversion of Ingested, EC_50_ = Effective Concentration, LC_50_ = Lethal Concentration, D = Diameter size of particle, SOR = Surfactant–Oil/Organic-Ratio, AFC_50_ = Antifeedant median Concentration, EO = Essential Oil, AI = Antifeedant Index, DC = Deterrence Concentration.

Antifeedant plant resources are mainly obtained from plant extracts and essential oil compounds. These resources are investigated in various forms, such as the active fractions of crude extract or essential oil (EO), and also in further processing screening steps to obtain isolation of pure active compounds. Essential oils are usually used as antifeedant resources in the stored and palm oil pest management, while crude plant extract is used as an antifeedant resource in crop management. Active compounds are not always more effective than crude extracts or essential oils; thus, choosing antifeedant resources is determined by the specificity and characteristics of the target insect pest. The active fractions of crude extract or essential oil (EO) may provide synergistic functional activity to the target insect pest. In addition, the utilization of antifeedant from crude extracts or essential oils offers a simpler process. In contrast, the application of pure active compounds has required sophisticated and high-cost production [39,74]. Therefore, the use of crude extracts or essential oils is interesting to explore in providing antifeedant plant resources.

It is known that grain insect pests are effectively controlled by EO due to their sensitivity to volatile semiochemicals [51,75]. Thus, antifeedant from essential oils is a preferable choice in stored grain pest management compared to plant extract. Contrary to this, antifeedant from plant extract shows higher efficacy for crop pest management compared to essential oils that are targeted against phytophagous insect pests [27]. Moreover, plant extract provides the possibility to form a solid paste extract that is suitable for residual application in oral targeting, allowing a great amount of residual antifeedant substance and persisting long enough to deter feeding activities [49]. Therefore, crude plant extract preparation has more opportunities to be applied for antifeedants in crop management.

The research progress of antifeedants on improving efficacy against target insect pests includes reservation and preparation techniques from conventional methods to the latest nanoformulas, as presented in Table 1. Many studies have reported on the advanced development of EO nanoformulation applied in stored pests and oil palm pest management. In contrast, rarely reported are studies on the nanoformulation of crude plant extract applied for insect pest crop management. Generally, before formulation, crude plant extracts are isolated to obtain pure active compounds. However, this route of preparation needs sophisticated and longer steps and, consequently, a reduced economic process. Few researchers propose to prepare a nanoformulation from a crude extract. However, the majority of reported studies are still in laboratory-scale production; only a few studies have reported on pilot-scale studies and field applications. Thus, given the great potential and abundant resources, the development of a nano-based formulation is promising in accelerating the applications of antifeedant; this is in line with the recommendations from Isman [7] on the prospect of antifeedant from plant resources. Despite many determining factors, the concern of relevant stakeholders is needed for the successful implementation of an antifeedant strategy for sustainable agriculture.

### 2.2. The Role of Nanotechnology in Plant-Derived Pesticide Formulations

**Figure 2:**
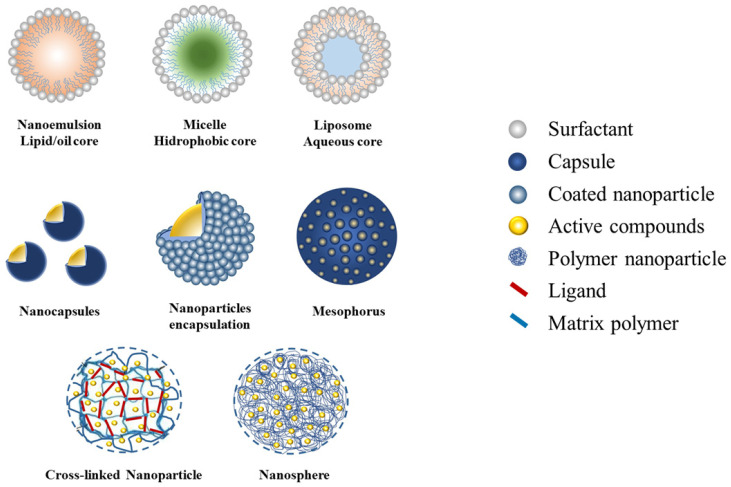
The types of nano-delivery biopesticide formulations.

Currently, nanotechnology is the breakthrough of various innovations in the development of bioinsecticide formulas [9,36]. Biopesticide formulas established through nanotechnology improve delivery performances and enhance their application efficiencies. It is well known that the smaller size of particles serves to increase the surface of the active ingredient and, consequently, improve the solubility. Moreover, the challenges involved are preparing the synthesis of the water-based medium, formula stability, mobility, and ensuring the delivery target system [76]. A broad variety of natural materials are used in the assembly of pesticide nanoformulations. There are two types of formulations—nano-particle pesticides and nano-carrier systems—to allow delivering active compounds to the target site. The structure of the delivery system includes the encapsulation of active compounds inside, a nanoparticulate polymeric shell, adsorption onto the nanoparticle surface, attachment onto the nanoparticle core via ligands, and entrapment within the polymeric matrix [77]. The properties of these various types of nanocarrier formulations are known to enhance the efficacy and efficiency of biopesticides against insect pests, i.e., a nanoemulsion loaded with essential oil from various plants products [78,79,80], plant extracts loaded in micelle with a hydrophobic core [58] and liposome with a hydrophilic core [81], as shown in Figure 2. Recently, materials from natural polysaccharides, proteins, alginates, silica, and other types of polymers have been utilized as nanoparticle encapsulants, such as chitosan, zein, gum arabic, and silica nanoparticles [31,78,82].

Botanical active compounds have also been reported to be successfully loaded in a nanocapsule and being mesoporous for the slow-release system as well as being entrapped in the matrix polymer and the cross-linked nanoparticles mediated by specific ligands [83,84,85,86]. It is well known that the characteristic content of organic active compounds inherent in botanical ingredients is that they are easily degraded and, consequently, have a lower long-term potency [12]. The various types of nanocarrier systems offer the appropriate properties to improve the efficacy and efficiency performance of plant-derived nano-pesticides’ delivery (Table 2).

Nano-emulsified carriers are emerging as the most intensively investigated of plant-derived pesticides (Table 2). This system is suitable to be adapted to EO and crude extracts of plant-derived pesticides by applying a simple emulsification method, requiring low energy and with suitable surfactants [23,92]. Emulsion-based formulations are designed to increase dispersion or solubility of ingredients, improve stability, and increase bioactivity and efficiency, especially in controlling insect crop pests [31]. Nanoemulsion formulas are extensively investigated for EO plant-derived nano-pesticides’ delivery to obtain desired properties due to the nano-sized droplet dispersion uniformity and the stability into two liquid phases by the fundamental role of the surfactants. Thus, the engineering characteristic and the properties of the delivery system can provide a slow-release performance [23]. Micelles are ideal nanocarriers for encapsulating, especially for insoluble-organic compounds such as plant extracts [93]. This allows the nano-sized insoluble-organic suspension dispersed in the water system that enhances the wettability and bioefficacy toward targeted insect pests [9]. Liposomes are vesicular to nanoscale structures, and which consist of a lipid bilayer covering an aqueous phase in the core [93]. The preparation of a liposomal nano-carrier has emerged as a promising aspect of nano-delivery biopesticides due to separate compartments that can encapsulate both the hydrophilic and hydrophobic active compounds that are effective against targeted insect pests [81].

The encapsulation involves a vesicular composed of the biodegradable matrix/polymer that encloses the active compounds in the inner core [9]. Nanocapsule and nanoparticle encapsulation increase the targeting delivery, and shell degrades slowly by environmental conditions, thus improving the chemical stability of organic compounds, such as volatile compounds commonly containing types of EO [93]. Mesoporous nanoparticles with hollow silica were adapted for water-soluble and lipid-dispersed controlled release biopesticide delivery systems. While nanospheres are designed as dense spherical vesicular systems in which active compounds are evenly distributed via adsorption or trapping in the nano-matrix/polymer, the cross-linked nanoparticles of the entrapped active compounds are mediated by ligands that act as sensors or markers for specific receptor molecules in targeted delivery. These efficient encapsulations and smart entrapped nano-carrier systems were confirmed to load the EO or pure active compounds with quite a high loading capacity with lethal and sublethal bioactivities due to a controlled slow-release mechanism [78,84,85,86,88].

Plant-derived nano-pesticides have been tailored for desired properties, involving the use of matrix types [94]. Studies have reported carrier systems prepared by organic and inorganic matrices/polymers and suitable surfactants as a means of delivering various extracts, EO, and their active compounds [88,94]. The utilization of nature/organic matrices’ resources matter is growing rapidly to compete with the non-organic matrices, such as chitosan, gum arabic, and zein. This carrier system maintains the susceptibility of active organic compounds to degradation so that they can be persistent for a longer period. Thus, these efficiently increase toxicity, fumigants, repellency, attractants, antifeedant, growth development, and oviposition inhibition [88,95].

The evaluation of studies shows that a compatible nanocarrier adopted in crude EO can even outperform or be comparable with the effectiveness of pure active compounds [78,89]. Nanocarrier biopesticide formulas can also enhance the effectiveness of pure active compounds to be comparable or more effective than synthetic insecticides in an in vitro bioassay test [81]. The performance of nanocarrier formulas of EO and plant extract can reduce the level of toxicity, indeed enhancing sublethal bioactivities such as the impact of antifeedant and repellency, and inhibiting growth regulation [58,88,91]. The advantages of the nanocarrier formula compared to conventional or synthetic insecticide formulas are determined through increased efficiency performance, such as the solubility and dispersion, formula stability, and release control mechanism offered by the nano-delivery system. This factor has a significant impact on increasing its efficacy against target insect pests. Plant-derived pesticides from abundant plant extracts resources are the most studied pesticides in the investigation of crop pest management. However, the potential compatibility of nanocarrier formulas for application is less explored.

Furthermore, the prominent role of the nano-delivery plant-derived pesticides formula is to reduce the level of toxicity so that the antifeedant and other potent sub-lethal bioactivities can be enhanced due to nano-delivery reserves. Especially for safety products in crop management, a plant-derived pesticides formula is hindered by toxicant residues and resistance problems. The challenges are compatibility with nanocarriers and resources for appropriate bioactivity on target insect pests and cost-effective formulation to allow the filed or practical application of recent advanced technological development.

### 2.3. Nano-Delivery System of Antifeedant Formulation

As antifeedant is potentially received from plant-derived bioactivity, it becomes an interesting object of study as an important component of integrated pest management, especially in crop pest insect control [4,96]. Further noted is that the antifeedant mode of action is determined by a feeding mechanism, which is induced by special taste receptors in insects that stop feeding activity. Antifeedants are generally obtained from the resources of plant extracts or essential oils that contain ingredients sensitive to insect taste receptors [46,97]. The biodiversity of potentially bioactive phytochemicals is the main source in formulating nanobiopesticides. Nanobiopesticides have been shown to have a significant impact on improving plant-derived pesticide properties, including antifeedant performance [27,36]. The efficiency and effectiveness of nanobiopesticides including antifeedants are enhanced by using nanoformulation polymers, metal oxides, active particles combined with micelles, etc. [36]. The last ten years of studies on nanoformula-based antifeedant investigations, involving the types of formulas, sources, methods, composition, and the performance of formulas as well as the antifeedants’ effectiveness against many insect pests, are summarized in Table 3.

Nanoparticle (NP) biopesticide formulas are currently in great demand for sublethal dose testing, including for antifeedant bioactivity [105]. As an example, the biosynthesis of silver nanoparticles using plant extracts [98,99,100] produces silver nanoparticles (AgNPs) through a simple and low-energy process. In general, the research purpose for metal nanoparticles is to find safer and lower concentration levels of cost-effective toxicants. Notably, only a few studies have reported the progress of a nanobiopesticide impacting on the formula’s efficiency, which is one of the important properties in the biopesticide nano-based formulation for application. It is emphasized that among those three examples [98,99,100] of inorganic nano-carriers (AgNPS) for the delivery of crude extract, generally, a nano-sized delivery system enhances bioactivity and suppresses toxicity compared to the control.

Another antifeedant nano-based formulation relies on a slow-release control designed to entrap the EO compounds by specific polymers, such as polymeric or chitosan nanoparticles [101,102,103]. The active biopesticide-based nanoparticle generally improves the efficiency of NPs in a controlled manner and shows prolonged bioactivity. However, the controlled activity established by the encapsulated structure does not necessarily contribute to any significant feed-deterrent activity of insect pests. By the treatments of EO-bergamot and EO-geranium, it displays antifeedant activities better than EO-PEG nanoparticles where the role of PEG encapsulation can improve loading efficacy by up to 75% against *Tribolium castaneum* [101]. This is in line with the treatments of the encapsulated neem oil in poly(ε-caprolactone) (PCL), poly (β–hydroxy-butyrate) (PHB), and poly (methylmethacrylate) (PMMA) polymeric nanoparticles compared to the broth neem oil against Spodoptera frugiperda. The observation result shows that only neem oil still provides antifeedant activity with a phagostimulant index < 1 at 7 days after spraying [102]. This is reasonable when considering that antifeedant activity is stimulated by a series of taste receptors as an impulse input to the insect feeding regulator. The encapsulation of the active ingredient must consider a matrix or polymer that accommodates the stimulate of the antifeedant compound when the polymer-enclosed material enters the oral and insect digestive system of the insect. This can be explained through the application of the chitosan-nanoparticle cross-linking agent formula studied by Zheng et al. [104]. The degree of polymeric encapsulant swelling is determined based on the pH value corresponding to the acidity level in the digestive system of *Solenopsis invicta* in correlation with the consuming activities. The cross-linked structure of polymers is not only appropriate for the slow-release of NP biopesticide but also for enhancing the efficacy and efficiency of the formula. Moreover, a clear explanation of the active ingredients’ absorption mechanism has been explored and reported. Interestingly, the advantage of this smart nanobiopesticide is that it can predict the impact, including the prolonged activity of active ingredients. Unfortunately, the practicality and cost of production constrain the large-scale field application of this kind of nanobiopesticide. Thus, improving the scale-up of production to meet field application remains a challenge.

A more practical and cost-effective antifeedant role model formula was displayed by a nano-based antifeedant formulation obtained from crude plant extract nanoparticle resources [40,58]. The neem gum nanosuspension can be prepared by a simple stirring method adding TiCl_4_ as a stabilizing agent on a certain composition, and has even tested as having a higher 100% antifeedant activity on *H. armigera* and *S. litura* larvae at a low concentration treatment (100 ppm) [40]. The insoluble organic extracts of *L. Camara* ethyl acetate nano-fraction can be dispersed in a water system by a simple reverse emulsion method with the composition of Tween 80 ratios as an appropriate surfactant [58]. The results show a significantly enhanced antifeedant activity in a strong category at LC_50_ value 0.39% concentration treatments against *Crocidolomia larvae*. However, the weakness of both formulas is easily agglomerated, hence it requires handling and agitation before application.

**Figure 3:**
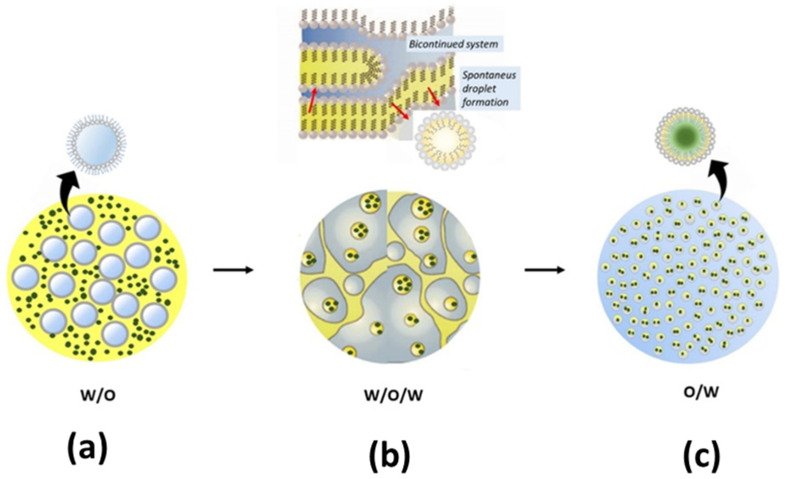
Dispersion of nanosuspensions in the water system by micelle formation (highlighted in blue-grey for water, yellow for oil/non-polar solvent, green for plant-extract suspension, and grey for surfactant/Tween 80): (**a**) water–oil (W/O) formation, (**b**) water–oil–water (W/O/W) formation adopted from McClements and Rao [107], and (**c**) oil–water (O/W) formation.

Despite the advantageous features of non-volatile active antifeedant resources, they are usually hindered by the characteristics of plant extract antifeedants to dissolve in water [9,106]. Furthermore, not all extracts are easy to dry to obtain a desirable nanopowder. Moreover, dispersing insoluble organic plant extract into nanosuspensions in the water system by the emulsification method is a breakthrough for obtaining nanosuspensions, as shown in Figure 3. Generally, micelles are formed due to the natural assembly properties of amphiphilic blocks’ surfactant in an aqueous medium; when the hydrophilic portion of the surfactant is added to the solution over the critical micelles concentration (CMC), the inner spherical micelles are formed into water [11] (Figure 3a). Thus, insoluble compounds are trapped in the core of micelle formation, which is called spontaneous emulsification (Figure 3b). The emulsion with micelle formation, as shown in Figure 3c, is also effective as a protective system of active ingredients with a one-layer surfactant. This formation does not require time for the encapsulant to dissolve when exposed to insect pests. Therefore, it can directly induce the phagostimulant deterrent receptors of phytophagous insect pests.

**Figure 4:**
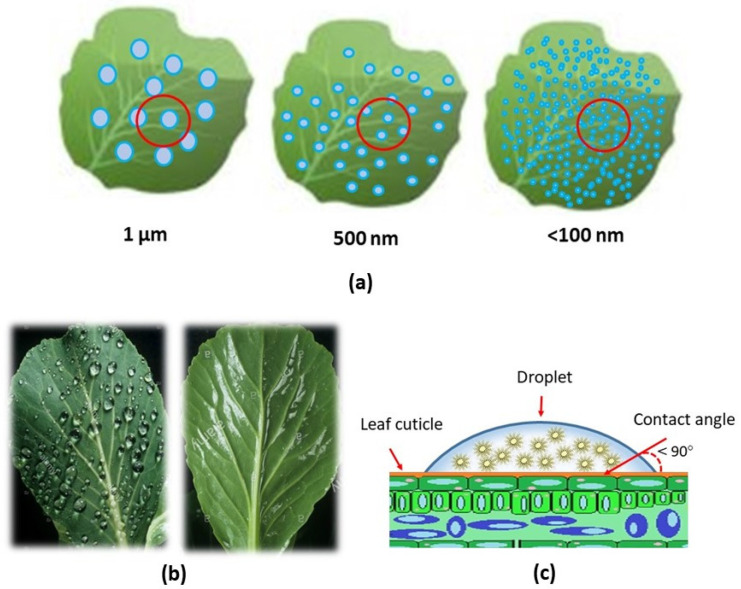
Illustration of the distribution of droplets containing nano-delivery system as a result of droplet size and surface wettability of leaf against droplets (**a**); the image comparison of nano-micelle droplet of low wettability (left) and high wettability (high) on the leaf surface (**b**); and the reduction in surface tension of the droplets containing nano-sized suspension on the leaf surface (<90°) (**c**).

The nano-delivery-based antifeedant is aimed at increasing the effectiveness and efficiency of active ingredients that are targeted and are safe for the environment. The inversion process that occurs during emulsification with the appropriate surfactant is known to disperse nano-sized suspensions in fine emulsion droplets [106,108,109]. The nanobiopesticide, which includes the antifeedant nano-delivery system, forms a stable dispersion, improves the efficacy and efficiency, and improves the wetting and spreading on the leaf surface [9]. In addition, antifeedant nanoparticles need to deposit and spread uniformly on the foliage surface, leading to increased retention rates and decreased spraying doses (Figure 4). Moreover, it is in line with that recommended by Zhao et al. [9] and Lade et al. [36], who state that the important aspects needed in the development of nanobiopesticides, especially in antifeedant nano-delivery strategies, are: (i) development of a water-based dispersion system, (ii) leaf-targeted deposition and dose transfer mechanism of nano-delivery, (iii) increased bioavailability mechanism of nano-based formulations, (iv) natural degradation and biosafety of residues. Moreover, advances in the application of nanomaterial formulation in pesticides have indicated that utilizing nanotechnology to design and prepare targeted pesticides with an environmentally responsive controlled release via chemical modifications and compounds offers great potential for creating new formulations [110,111].

This antifeedant delivery by micelle formulation is still considered premature to accommodate the abovementioned desired properties. There are still many limitations within the study on the efficiency that need to be investigated. The challenges are how to evaluate the effectiveness of its efficiency when interacting with UV exposure, the material persistence, the stability of efficacy performance during field application, as well as the side effects on non-target organisms. However, the development of this antifeedant formula offers a bright prospect for alternative formulas received from plant extract resources. Fortunately, there are abundant available resources of plant extracts and they can be prepared with a simple method, low costs, and easy handling, creating a forthcoming insight for the field-scale application of this nano-delivery antifeedant. Considering another important aspect of integrated pest management, antifeedant bioactivity plays an important part and should be integrated with other approaches in phytophagous insect pest control. It allows the anticipation of insect resistance with multiple modes of action such as antifeedant activity, growth and development inhibition, anti-oviposition, reduced fecundity, and repellency.

## 3. Summary

The effectiveness of antifeedant strategies is determined by the specific active functionality of the relevant antifeedant resources to the characteristics of the target insect pest. The preparation of crude plant extract by nano-based formulation potentially enhances the efficacy and efficiency of antifeedant applications for controlling crop insect pests. Crude plant extracts are potentially more economical antifeedant resources because they require simple steps of processing and synthesis; consequently, they are potentially an important part of crop management. The structure of nano-delivery plant-derived pesticides, including nanoemulsions, micelles, liposomes, encapsulation, mesoporous nature, and cross-linking, offer enhanced efficacy and efficiency performance against insect pests, such as by displaying solubility and dispersion, formula stability, and a release control mechanism.

The antifeedant nano-delivery system can increase sublethal bioactivity in both crude extracts/EOs and pure compounds. The functional groups of antifeedant molecules in nanoformulations do not change, thus their biological activity remains as antifeedants [58]. As the formulation is a nano-sized emulsion, it provides a higher surface area, leading to enhancing the biological activity of the antifeedant molecule. The nanoemulsion could be uniformly deposited on the surface of the leaf; therefore, there is a higher possibility of the pest consuming the leaf containing the antifeedant molecule [9,58]. Fortunately, the diversity of plant extracts and their abundance become a great potential application in crop pest management. However, there remain challenges regarding formulation-related preparation and their functionalities, including the compatibility of nanocarriers with the active compound of plant extracts. Thus, it is important for the investigation of nano-delivery plant extracts to have proper bioactivity in target insect pests, cost-effective formulations, and practical applications. In the case of a micelle-structure nano-delivery system, improvement is introduced by the enhancement of antifeedant activity and improving the wettability to create a uniform distribution on the leaf surface. Moreover, this formulation provides direct induction to the phagostimulant deterrent receptors of phytophagous insects without being hindered by the degradation process, which usually occurs in another formula, polymeric encapsulant.

## 4. Future Direction

Antifeedant nano-based delivery systems offer the opportunity for application via the utilization of plant-derived pesticides, especially plant-extract resources, in insect crop pest management. Nanotechnology takes an important role in the development of antifeedant nano-based delivery systems. Thus, it is emphasized that the development of antifeedant nano-delivery strategies includes: (i) development of a water-based dispersion system, (ii) leaf-targeted deposition and dose transfer mechanism of nano-delivery, (iii) increased bioavailability mechanism of nano-based formulations, (iv) natural degradation and biosafety of residues. Finally, it is important to encourage antifeedant application for crop insect pest management because this provides many advantages for sustainable agriculture goals. It is concluded that the nano-delivery antifeedant from plant extracts creates a forthcoming insight for field-scale application as a result of the economic production process.

## Figures and Tables

**Table 2:** The plant-derived nano-pesticides’ delivery.

No.	Carrier System	Preparation and Matrix/Polymers, Surfactant	Properties	Active Compounds	Efficacy/Bioactivities	Ref.
1	Nanoemulsion	Oil-in-water by low energy emulsification produce nanoemulsion loaded by EO sea fennel in core (EO:Tween 20 = 1:3)	D = 50–70 nm ZP = −18.3 mV	Seed sea fennel essential oil (EO) (dillapiole and γ-terpinene)	Nanoemulsion enhances the toxicity, inhibits longevity and fecundity of *Aedes aegypti* and *Spodoptera litura.* Nanoemulsion is more effective compared to crude EO but less effective compared to pure EO	[78]
2	Nanoemulsion	Oil-in-water nanoemulsion loaded by EO *Mentha spicata* in core (EO:Tween 80 = 1:1)	D = 97.8 nm EE = 52.0%	*Mentha spicata* EO with major compounds 8-cineole (19.55%) and menthol acetate (14%)	The higher larvicidal toxicity against *Culex pipiens* and *Musca domestica* larvae compared with the normal mint oil and lambda-cyhalothrin (synthetic)	[79]
3	Nanoemulsion	Oil-in-water by spontaneous emulsification produce nanoemulsion loaded by EO in core (EO:Tween 80 = 3:1)	D = 131.37 ± 0.29 nm ZP = −30 mV	Sweet orange EO with major monoterpene compounds (R-limonene, β-myrcene, α-pinene, linalool, and sabinene)	Enhance repellency, fumigant, and acute toxicity against *Tribolium confusum* and *Cryptolestes ferrugineus*	[87]
4	Micelle	Droplet emulsion loaded by ethyl acetate fraction (EAF) *Lantana camara* nano-suspension (EAF:Tween 80 = 3:2)	D = 8.3 ± 1.3 nm, ZP = −8.9 mV	Saponins, alkaloids, and steroids	Moderate toxicity and strong antifeedant activity against *Crocidolomia pavonana* lavae	[58]
5	Liposome	*Ruta graveolens* dichloromethane extract encapsulate in chitosan nanoemulsions and lipid nanosystems (liposomes) with ethanolic injection and thin-film hydration	Liposomes ethanolic D = 121 nm, PI = 0.256, EE = 93% Liposomes thin-film hydration D = 203 nm, PI = 0.185, EE = 73%	Dichloromethane extract of *Ruta graveolens* (quinolin, benzopyran, and acridone derivatives)	Cytotoxic activity against *Spodoptera frugiperda* (Sf9) insect cells is more effective than chlorpyrifos (synthetic insecticides)	[81]
6	Nanocapsules	Nanocapsules formed by chitosan precipitation loaded with *Achillea millefolium* essential oil (AEO)	D = 85–145 nm	Groups of terpenes: 1,8-cineol, camphor, borneol, phellandrene, and linalool-L	Improving the fumigant effectiveness and efficiency by slow and persistent release against the *A. millefolium* L.	[83]
7	Nanocapsules	Nanocapsules formed by SiO_2_ precipitation loaded by sea fennel EO (SiNPs)	D = 20–78 nm ZP = −11.7 to 34.3 mV	Seed sea fennel essential oil (EO) (dillapiole and γ-terpinene)	Higher toxicity, reduced longevity, and fecundity compared with the nanoemulsion and pure essential oil	[78]
8	Nanoparticles encapsulation	Chitosan nanoparticles (CSNPs) loaded by *Piper nigrum* essential oil (PNO)	D = 527.5 nm	*Piper nigrum* essential oil (PNO)	Toxicity activity, fumigant activity against *Sitophilus oryzae* and *Tribolium castaneum.*	[82]
9	Nanoparticles encapsulation	Zein nanoparticles loaded with geraniol	D = 172.3 ± 3.8 nm, PI = 0.351 ± 0.032, ZP = −12 to −25 mV, EE > 90%	Geraniol and R-citronellal	Decreasing toxicity and phytotoxicity but enhanced repellency against *Tetranychus urticae* mite.	[88]
10	Nanoparticles encapsulation	Chitosan and gum arabic nanoparticles containing geraniol	D = 200−300 nm, PI = 0.21−0.78, ZP = −21 to −35 mV, EE = 91−98%	Geraniol	Significant attraction activity (attractant) against whitefly (*Bemisia tabaci*)	[88]
11	Mesoporous	Hollow mesoporous silica (HMS) nanoparticles, using carboxylated β-cyclodextrin (CD) as a capping molecule HMS with Si–NH_2_ as gatekeeper (HMS–NH_2_), HMS with β-cyclodextrin (β-CD) as gatekeeper (HMS-CD)	HMS (D = 150.16 nm, PI = 0.036, ZP = −2.47 mV) HMS-NH_2_ (D = 153 nm, PI = 0.041, ZP = 7.21 mV) IDC loaded HMS–NH_2_ (D = 153.78 nm, PI = 0.034, ZP = −19.7 mV) IDC loaded HMS-CD (D = 193.26 nm, PI = 0.011) HMS average pore size 2.41 nm Loading efficiency 26.42%	Indoxacarb (IDC) carboxylated β-cyclodextrin	Toxic activity against *Spodoptera frugiperda*	[84]
12	Mesoporous	Mesoporous silica (MCM) nanoparticles modified by salicylaldimine (Sal-MCM), furfuralimine (Fur-MCM), and benzaldehyde imine (Ben-MCM)	Me (D = not available, ZP = −38.82 mV, SR (10 h) = 70.82%) MCM (D = 833 ± 11 nm, ZP = −20.16 mV, SR (10 h) = 82.88%) Sal-MCM (D = 789 ± 12 nm, ZP = 15.77 mV, SR (10 h) = 48.59%) Fur-MCM (D = 701 ± 12 nm, ZP = 29.89 mV, SR (10 h) = 56.63%) Ben-MCM (D = 763 ± 12 nm, ZP = 25.70 mV, SR (10 h) = 37.21%)	Methyl eugenol (Me)	Attraction activity against *Bactrocera dorsalis* The best formula shows the highest lure rate of Fur-MCM loading by Me equals 73% of the pure Me	[89]
13	Mesoporous	Mesoporous silica nanoparticles (MSNs) from tetraethyl orthosilicate (TEOS) hydrolysis modified by cinnamon oil encapsulated with silica nanoparticles (CESN)	Spherical silica nanoparticles, well dispersed in water, provide a maximal interface to load optimal cinnamon oil for the delivery target that induces the biological mechanism indicated by protein profiles	Cinnamon oil	Insect pest *Corcyra cephalonica* LC_50_ MSNs Total protein content = 28.88 mg mL^−1^ Inhibition (pupa = 47.50%, adult emergence = 28%) LC_50_ CESN Total protein content = 28.65 mg mL^−1^ Inhibition (pupa = 45.0%, adult emergence = 0%) LC_50_ cinnamon oil Total protein content = 28.2 mg mL^−1^ Inhibition (pupa = 37.7%, adult emergence = 0%) Control (−) untreated Total protein content = 32.56 mg mL^−1^ Inhibition (pupa = 90.0%, adult emergence = 87.5%) Control (+) silica gel Total protein content = 30.32 mg mL^−1^ Inhibition (pupa = 47.5%, adult emergence = 78.0%)	[90]
14	Cross-linked nanoparticle	Chitosan nanoparticles (CSNPs) cross-linked by ionic gelation of sodium tripolyphosphate (TPP), coating EO (1% Tween 80)	D < 563.3 nm, ZP = −12.12 mV) EE = 70% Loading capacity > 12.31%.	Peppermint essential oil: L-menthone (32.27%) menthol (23.47%)	The higher toxicity by the mechanism of acetylcholinesterase inhibition on *S. oryzae* and *T. castaneum*	[86]
15	Cross-linked nanoparticle	Chitosan nanoparticles (CSNPs) cross-linked by glutaraldehyde (GLA) and tripolyphosphate (TPP)	Electron micrograph measured: CSNPs (D = 32–90 nm) DLS measured: CSNPs-TPP-PONEEM (D = 122.7 nm, PI = 0.282, EE = 59.34%) CSNPs-GLA/TPP-PONEEM (D = 243.5 nm, PI = 0.57, EE = 65%)	Chitosan and azadirachtin	Effective as antifeedant, larvicidal, and growth-regulating activities, at very low concentrations	[91]
16	Nanosphere	Encapsulated *Zanthoxylum riedelianum* fruit EO in poly-ε-caprolactone (PCL) nanospheres	D = 106.7–129.2 nm, pH 6, ZP = < −19.0 mV, EE > 98%	*Z. riedelianum* fruit EO Major compounds: limo-nene (29.22%), β-myrcene (22.79%), bicyclogermacrene (18.13%)	Effective as oviposition deterrent and low toxicity against nymphs of *Bemisia tabaci*	[85]

Note: D = droplet/particles size, ZP = zeta potential, EE = encapsulation efficiency, SR = sustain release.

**Table 3:** Antifeedant by nano-based formulation.

No.	Nanoparticles/Nanocarriers	Resources and Compounds	Methods	Compositions	Properties and Performances	Antifeedant Activities	Ref.
1	Silver nanoparticles	*Manilkara zapota* leaf aqueous crude extract	Chemical reaction of *M. zapota* leaf extract and AgNO_3_	*Manilkara zapota* leaf aqueous crude extract = 12 mL Aqueous 1 mM AgNO_3_ = 88 mL	D = 70–140 nm, nano-size enhances bioactivity and reduces toxicity compared to control	Insect pest *M. domestica* Positive control: dichlorvos The feeding deterrent activity (3 h exposure): *Crude extracts* LD_50_ = 28.35 mg mL^−1^, LD_90_ = 89.19 mg mL^−1^ *Synthesized AgNPs* LD_50_ = 3.64 mg mL^−1^, LD_90_ = 7.74 mg mL^−1^	[98]
2	Silver nanoparticles	*Aristolochia indica * leaf aqueous extract	Chemical reaction of *A. indica* leaf extract and AgNO_3_	*Aristolochia indica* leaf aqueous extract = 15 mL Aqueous 3 mM AgNO_3_ = 85 mL	D = 112.35 nm, nano-size enhances bioactivity and reduces toxicity compared to control	Insect pest *H. armigera* larvae Positive control: azadirachtin pure compounds Negative control: Aqueous extract Synthesized Ag NPs Strong antifeedant (92.40%, LC_50_ 365.72 mg mL^−1^) *Azadirachtin pure compounds* Strong antifeedant (97.28%, LC_50_ 348.98 mg mL^−1^) *Aqueous extract* Good antifeedant (72.22%, LC_50_ 623.3 mg mL^−1^)	[99]
3	Silver nanoparticles	*Leonotis nepetifolia* leaf aqueous extract	The chemical reaction of *L. nepetifolia* leaf extract and AgNO_3_	*Aristolochia indica* leaf aqueous extract = 2.5 mL Aqueous 1 mM AgNO_3_ = 47.5 mL	AgNPs D = 37.5 nm (monodisperse and spherical structures)	Insect pest *S. litura* and *H. armigera* Positive control: azadirachtin pure compounds Negative control: *L. nepetifolia* plant extract *AgNPs* Strong antifeedant activities (FDI) at 150 ppm 78.77% against *S. litura* 82.16% against *H. armigera* *Azadirachtin pure compounds* Strong antifeedant activities (FDI) at 150 ppm 87.43% against *S. litura* 90.47% against *H. armigera* *L. nepetifolia* plant extract moderate antifeedant activities (FDI) at 150 ppm 48.17% against *S. litura* 50.92% against *H. armigera*	[100]
4	Essential oil (EO) nanoparticles	Geranium EO (contain citronellol and geraniol) or Bergamot EO (contain linalyl acetate), and polyethylene glycol (PEG) 6000 as surfactant	Melt-dispersion method	The ratio of essential oil (geranium EO or bergamot EO) to PEG (10%)	Geranium EO-PEG NPs D < 235 nm, PI = 0.280 loading efficacy = 75% Bergamot EO-PEG NPs D < 184 nm, PI = 0.309 loading efficacy = 71%	Insect pest *Tribolium castaneum* and *Rhizopertha dominica* Negative control: geranium EO and bergamot EO *Geranium EO-PEG NPs* Antifeedant activities (FDI) 60% against *T. castaneum* 48% against *R. dominica* *Geranium EO* Antifeedant activities (FDI) 11% against *T. castaneum* 57% against *R. dominica* *Bergamot EO-PEG NPs* Antifeedant activities (FDI) 54% against *T. castaneum* 39% against *R. dominica* *Bergamot EO* Antifeedant activities (FDI) 3% against *T. castaneum* 83% against *R. dominica*	[101]
5	Polymeric nanoparticles	Active compounds: the neem (*Azadirachta indica*) oil and neem seed kernel extract The polymers: Poly (ε-caprolactone) (PCL), poly (β–hydroxy-butyrate) (PHB), poly (methylmethacrylate) (PMMA)	Colloidal suspensions prepared by interfacial preformed polymer deposition Spray drying of colloidal suspensions	Solution polyvinyl alcohol (PVA) 0.5 mL of benzyl-benzoate containing 12.5 mg of neem oil phospholipids (250 mg) 0.25, 0.5, and 0.75 g PCL (Aldrich) and PHB (Fluka) dissolved in PVA 25 mL 0.25 g PMMA (Aldrich) dissolved in PVA 25 mL	Efficiencies nanocapsules (68%) nanospheres (33%)	Insect pest *Spodoptera frugiperda* Negative control: neem oil The nanoformulations Antifeedants at 1st DAS: [P]NC-PCL (0.25) Preference Index (PI) 0.77 [S]NS-PHB (0.25) Preference Index (PI) 0.77 neem oil Preference Index (PI) 0.59 Antifeedants at 3rd DAS [P]NC-PCL (0.25) Preference Index (PI) 0.81 [S]NC-PMMA (0.25) Preference Index (PI) 0.88 neem oil Preference Index (PI) 0.90 Antifeedants at 7th DAS [S]NS-PHB (0.25) Preference Index (PI) 0.50 neem oil Preference Index (PI) 0.89	[102]
6	Polymeric nanoparticles	Essential oils (EOs) from peppermint and palmarosa Essential oil loaded polymeric nanoparticles (EOPN) Polymer: Polyethylene glycol 6000	Melt-dispersion method	Solvent ethanol 20 g PEG and 2 g EO	7 days post-formulation: EOPN-peppermint: D = 310 nm, PI = 0.61, LE = 93.75% EOPN-palmarosa: D = 203 nm, PI = 0.16, LE = 89.7%	Insect pest German cockroach *Blatella germanica L.* At doses of 4 mg disc^−1^, *EOPN-palmarosa* highly antifeedant (FDI = 76.9%) *EOPN-peppermint* highly antifeedant (FDI = 76.5%)	[103]
7	Polymeric nanoparticles	*Piper nigrum* essential oil (PNO) Chitosan nanoparticles (CS/PNO NPs) Polymer: Chitosan and sodium tripolyphosphate (TPP) as cross-linking agent	Emulsification method and ionic gelation	Total solution = 40 mL Chitosan solution 1% *w*/*v* + acetic acid solution 1% *v*/*v* Tween 80 (0.45 g) PNO (0.81 g) + dichloromethane (4 mL), TPP (4% *w*/*v*)	CS/PNO NPs D = 527.5 nm, ZP = −5.34 mV, LE (40.62 ± 0.26)%	Insect pest *S. oryzae* and *T. castaneum* Negative control: acetone CS/PNO NPs High antifeedant FDI 100% against *S. oryzae* and *T. castaneum*	[82]
8	Polymeric nanoparticles	PONNEEM^®^ (neem oil, karanj oil, azadirachtin, and karanjin), Chitosan nanoparticles CSNs-TPP-PONNEEM NPs and CSNs-GLA-PONNEEM NPs Polymer: Chitosan and sodium tripolyphosphate (TPP) as cross-linking agent and glutaraldehyde (GLA)	Ultrasonic-added ionic gelation freeze-drying method	PONNEEM^®^ 41% neem oil, 41% karanj oil, 1% azadirachtin, 1% karanjin, 13% emulsifier and stabilize Chitosan in 1% acetic acid (50 mg/50 mL), TPP or GLA (50 mg/50 mL)	CSNs-TPP-PONNEEM NPs (D = 122.7 nm; PI = 0.282, encapsulation efficiencies = 59.34%) CSNs-GLA-PONNEEM NPs (D = 243.5 nm; PI = 0.57, encapsulation efficiencies = 65%)	Insect pest *H. armigera* larvae Positive control: PONNEEM Negative control: CSNs-TPP and CSNs-GLA PONNEEM Antifeedant activities 100%; at 0.3% CSNs-TPP-PONNEEM Strong antifeedant activity (88.5%) CSNs-GLA-PONNEEM Medium antifeedant activity (72.3%) CSNs-TPP Medium antifeedant activity (76.4%) CSNs-GLA Medium antifeedant activity (65.3%)	[91]
9	Polymeric nanoparticles	Nanoparticles synthesized from chitosan and carbo-xymethyl chitosan Carboxymethyl chitosan (CS/CMCS-NPs) Cross-linking agent: amino groups glutaraldehyde (GA)	Emulsion chemical cross-linking method with ultrasonic-aided ionic gelation	0.01% and 0.02% CS/CMCS-NPs, 5 mL of 0.4% CMCS + CS/CS-NPs (1.5 mL of 0.5% glutaralde-hyde solution (GA) + 5 mL 0.3% CS)	SEM (D = 30–50 nm) PSA (D = 142.1 ± 2.0 nm, PI = 0.171 ± 0.002)	Insect pest *Solenopsis invicta* *After 4 days of treatments* 0.01% CS/CMCS-NPs S-NPs antifeedant activity (40%) 0.02% CS/CMCS-NPs S-NPs antifeedant activity (50%) *After 16 days of treatments* 0.01% CS/CMCS-NPs S-NPs antifeedant activity (60%) 0.02% CS/CMCS-NPs S-NPs antifeedant activity (80%)	[104]
10	Polymeric nanoparticles	The extract neem gum (NGE) powder (the majority of oleic acid compounds 31.45%) Neem gum nano formulation (NGNF)	Simple mixing method	Neem gum aqueous suspension (0.5% *w*/*v*) TiCl_4_ (stabilizing agent) with mixing ratio of 5:95, 10:90, 15:85, 20:80, and 25:75 mL	D = 20–40.83 nm and the average size of 31.27 nm	Insect pest *H. armigera* and *S. litura* larvae. Positive control: azadiractin NGNF at 100 ppm Strong antifeedant activity 100% against *H. armigera* and *S. litura* larvae. NGE at 100 ppm Medium antifeedant activity 74.82% against *H. armigera* 82.21% against *S. litura* larvae. Azadiractin at 100 ppm Medium antifeedant activity 68.26% against *H. armigera* 76.80% against *S. litura* larvae.	[40]
11	Micelle	Ethyl acetate fraction of *Lantana camara* (saponins, alkaloids, and steroids) Surfactant = Tween 80	Low energy phase inverse method aided sonication with the variation of surfactant–organic ratio (SOR)	Ethyl acetate fraction (EAF) *Lantana camara* (0.55% *w*/*v*) in aquadest Tween 80:EAF or SOR 1:11	D = 8.3 ± 1.3 nm, distribution 77%, lowest contact angles (48.5°) on the cabbage leaf surface	Insect pest *C. pavonana* larvae Negative control: EAF pre-emulsion EAF nanosuspension SOR 11 strong antifeedant category LC_50_ (0.39%) EAF pre-emulsion weak antifeedant category LC_50_ (0.69%)	[58]

## Data Availability

Not applicable.

## References

[1] FAO Pesticides Use, Pesticides Trade and Pesticides Indicators. Global, Regional and Country Trends, 1990–2019. 2021. FAOSTAT Analytical Brief Series No. 29. Rome. https://www.fao.org/3/cb6034en/cb6034en.pdf..

[2] Kilani-Morakchi S., Morakchi-Goudjil H., Sifi K. (2021). Azadirachtin-Based Insecticide: Overview, Risk Assessments, and Future Directions. Front. Agron..

[3] Karuppuchamy P., Venugopal S. (2016). Integrated Pest Management.

[4] Lengai G.M.W., Muthomi J.W., Mbega E.R. (2020). Phytochemical activity and role of botanical pesticides in pest management for sustainable agricultural crop production. Sci. Afr..

[5] Damalas C.A., Koutroubas S.D. (2018). Current Status and Recent Developments in Biopesticide Use. Agriculture.

[6] Rani L., Thapa K., Kanojia N., Sharma N., Singh S., Grewal A.S., Srivastav A.L., Kaushal J. (2021). An Extensive Review on the Consequences of Chemical Pesticides on Human Health and Environment. J. Clean. Prod..

[7] Isman M.B. (2020). Botanical Insecticides in the Twenty-First Century—Fulfilling Their Promise?. Annu. Rev. Èntomol..

[8] Sharma A., Shukla A., Attri K., Kumar M., Kumar P., Suttee A., Singh G., Barnwal R.P., Singla N. (2020). Global trends in pesticides: A looming threat and viable alternatives. Ecotoxicol. Environ. Saf..

[9] Zhao X., Cui H., Wang Y., Sun C., Cui B., Zeng Z. (2018). Development Strategies and Prospects of Nano-based Smart Pesticide Formulation. J. Agric. Food Chem..

[10] Kala S., Sogan N., Agarwal A., Naik S., Patanjali P., Kumar J., Egbuna C., Sawicka B. (2020). Biopesticides formulations and delivery techniques. Natural Remedies for Pest, Disease and Weed Control.

[11] Nuruzzaman M., Rahman M.M., Liu Y., Naidu R. (2016). Nanoencapsulation, Nano-guard for Pesticides: A New Window for Safe Application. J. Agric. Food Chem..

[12] De Oliveira J.L., Campos E.V.R., Fraceto L.F. (2018). Recent Developments and Challenges for Nanoscale Formulation of Botanical Pesticides for Use in Sustainable Agriculture. J. Agric. Food Chem..

[13] Mittal D., Kaur G., Singh P., Yadav K., Ali S.A. (2020). Nanoparticle-Based Sustainable Agriculture and Food Science: Recent Advances and Future Outlook. Front. Nanotechnol..

[14] Gasic S., Tanovic B. (2013). Biopesticide formulations, possibility of application and future trends. Pestic. Fitomedicina.

[15] Chin C.-P., Lan C.-W., Wu H.-S. (2012). Application of biodiesel as carrier for insecticide emulsifiable concentrate formulation. J. Taiwan Inst. Chem. Eng..

[16] Patzke H., Schieber A. (2018). Growth-inhibitory activity of phenolic compounds applied in an emulsifiable concentrate-ferulic acid as a natural pesticide against *Botrytis cinerea*. Food Res. Int..

[17] Knowles A. (2007). Recent developments of safer formulations of agrochemicals. Environmentalist.

[18] Pirzada T., de Farias B.V., Mathew R., Guenther R.H., Byrd M.V., Tim L., Sit T.L., Pal L., Opperman C.H., Khan S.A. (2020). Recent advances in biodegradable matrices for active ingredient release in crop protection: Towards attaining sustainability in agriculture. J. Colloid Interface Sci..

[19] Waghmare J.T., Ware A.M., Momin S.A. (2007). Neem oil as pesticide. J. Dispers. Sci. Technol..

[20] Puripattanavong J., Songkram C., Lomlim L., Amnuaikit T. (2013). Development of concentrated emulsion containing nicotiana tabacum extract for use as a pesticide. J. Appl. Pharm. Sci..

[21] Shao H., Xi N., Zhang Y. (2018). Microemulsion formulation of a new biopesticide to control the diamondback moth (Lepidoptera: Plutellidae). Sci. Rep..

[22] Pavela R., Benelli G., Pavoni L., Bonacucina G., Cespi M., Cianfaglione K., Bajalan I., Morshedloo M.R., Lupidi G., Romano D. (2019). Microemulsions for delivery of Apiaceae essential oils—Towards highly effective and eco-friendly mosquito larvicides?. Ind. Crops Prod..

[23] Pavoni L., Benelli G., Maggi F., Bonacucina G. (2019). Green Nanoemulsion Interventions for Biopesticide Formulations.

[24] Palermo D., Giunti G., Laudani F., Palmeri V., Campolo O. (2021). Essential Oil-Based Nano-Biopesticides: Formulation and Bioactivity against the Confused Flour Beetle *Tribolium confusum*. Sustainability.

[25] Isman M. (2002). Insect antifeedants. Pestic. Outlook.

[26] Isman M.B. (2006). Botanical insecticides, deterrents, and repellents in modern agriculture and an increasingly regulated world. Annu. Rev. Entomol..

[27] Miresmailli S., Isman M.B. (2014). Botanical insecticides inspired by plant–herbivore chemical interactions. Trends Plant Sci..

[28] Koul O. (2005). Insect Antifeedants.

[29] Koul O. (2016). Antifeedant Phytochemicals in Insect Management (so Close yet so Far). Ecofriendly Pest Management for Food Security.

[30] Usman M., Farooq M., Wakeel A., Nawaz A., Alam Cheema S.A., Rehman H.U., Ashraf I., Sanaullah M. (2020). Nanotechnology in agriculture: Current status, challenges and future opportunities. Sci. Total. Environ..

[31] Chaud M., Souto E.B., Zielinska A., Severino P., Batain F., Oliveira J., Alves T. (2021). Nanopesticides in agriculture: Benefits and challenge in agricultural productivity, toxicological risks to human health and environment. Toxics.

[32] Mustafa I.F., Hussein M.Z. (2020). Synthesis and technology of nanoemulsion-based pesticide formulation. Nanomaterials.

[33] Oliveira J.L., Campos E., Bakshi M., Abhilash P., Fraceto L. (2014). Application of nanotechnology for the encapsulation of botanical insecticides for sustainable agriculture: Prospects and promises. Biotechnol. Adv..

[34] Shivanandappa T., Rajashekar Y. (2014). Mode of action of plant-derived natural insecticides. Advance in Plant Biopesticides.

[35] Kumar S., Nehra M., Dilbaghi N., Marrazza G., Hassan A.A., Kim K.-H. (2019). Nano-based smart pesticide formulations: Emerging opportunities for agriculture. J. Control. Release.

[36] Lade B.D., Google D.P., Lade D.B., Moon G.M., Nandeshwar S.B., Kumbhare S.D. (2019). Nanobiopesticide Formulations: Application Strategies Today and Future Perspectives.

[37] Athanassiou C.G., Kavallieratos N.G., Benelli G., Losic D., Rani P.U., Desneux N. (2018). Nanoparticles for pest control: Current status and future perspectives. J. Pest Sci..

[38] Saini R.K., Patel S., Bajpai J., Bajpai A.K., Rakhimol K.R., Thomas S., Volova T., Jayachandran K. (2020). Advanced controlled nanopesticide delivery systems for managing insect pests. Controlled Release of Pesticides for Sustainable Agriculture.

[39] Forim M.R., Costa E.S., Silva M.F.D.G.F.D., Fernandes J.B., Mondego J.M., Junior A.L.B. (2013). Development of a New Method To Prepare Nano-/microparticles Loaded with Extracts of *Azadirachta indica*, Their Characterization and Use in Controlling *Plutella xylostella*. J. Agric. Food Chem..

[40] Kamaraj C., Gandhi P.R., Elango G., Karthi S., Chung I.-M., Rajakumar G. (2018). Novel and environmental friendly approach; Impact of Neem (*Azadirachta indica*) gum nano formulation (NGNF) on *Helicoverpa armigera* (Hub.) and *Spodoptera litura* (Fab.). Int. J. Biol. Macromol..

[41] Thomine E., Mumford J., Rusch A., Desneux N. (2021). Using crop diversity to lower pesticide use: Socio-ecological approaches. Sci. Total Environ..

[42] Baker B.P., Green T.A., Loker A.J. (2020). Biological control and integrated pest management in organic and conventional systems. Biol. Control.

[43] Rezaei R., Safa L., Ganjkhanloo M.M. (2020). Understanding farmers’ ecological conservation behavior regarding the use of integrated pest management—An application of the technology acceptance model. Glob. Ecol. Conserv..

[44] Jiang X., Hansen H.C.B., Strobel B.W., Cedergreen N. (2018). What is the aquatic toxicity of saponin-rich plant extracts used as biopesticides?. Environ. Pollut..

[45] Koul O. (2008). Phytochemicals and Insect Control: An Antifeedant Approach. Crit. Rev. Plant Sci..

[46] Purrington C. (2017). Antifeedant Substances in Plants. Encycl. Appl. Plant Sci..

[47] Bruce T.J.A., Smart L.E., Birch A.N.E., Blok V.C., MacKenzie K., Guerrieri E., Cascone P., Luna E., Ton J. (2016). Prospects for plant defence activators and biocontrol in IPM: Concepts and lessons learnt so far. J. Crop Prot..

[48] Sandjo L.P., Kuete V. (2013). Triterpenes and Steroids from the Medicinal Plants of Africa.

[49] Suckling D.M., Conlong D.E., Carpenter J.E., Bloem K.A., Rendon P., Vreysen M.J.B. (2017). Global range expansion of pest Lepidoptera requires socially acceptable solutions. Biol. Invasions.

[50] Kłyś M., Malejky N., Nowak-Chmura M. (2017). The repellent effect of plants and their active substances against the beetle storage pests. J. Stored Prod. Res..

[51] Lucia A., Guzmán E. (2021). Emulsions containing essential oils, their components or volatile semiochemicals as promising tools for insect pest and pathogen management. Adv. Colloid Interface Sci..

[52] Stanley V., Hickerson K., Daley M. (2012). Supermarket Supply Chains in Horticulture in India: The Novel Marketing Models, Effects and Policies. Agrotechnology.

[53] Urrutia R.I., Yeguerman C., Jesser E., Gutierrez V.S., Volpe M.A., González J.O.W. (2021). Sunflower seed hulls waste as a novel source of insecticidal product: Pyrolysis bio-oil bioactivity on insect pests of stored grains and products. J. Clean. Prod..

[54] Rajkumar V., Gunasekaran C., Christy I.K., Dharmaraj J., Chinnaraj P., Paul C.A. (2019). Toxicity, antifeedant and biochemical efficacy of *Mentha piperita* L. essential oil and their major constituents against stored grain pest. Pestic. Biochem. Physiol..

[55] Kiran S., Prakash B. (2015). Assessment of Toxicity, Antifeedant Activity, and Biochemical Responses in Stored-Grain Insects Exposed to Lethal and Sublethal Doses of *Gaultheria procumbens* L. Essential Oil. J. Agric. Food Chem..

[56] Wang C.-F., You C.-X., Yang K., Guo S.-S., Geng Z.-F., Fan L., Du S.-S., Deng Z.-W., Wang Y.-Y. (2015). Antifeedant activities of methanol extracts of four *Zanthoxylum* species and benzophenanthridines from stem bark of *Zanthoxylum schinifolium* against *Tribolium castaneum*. Ind. Crops Prod..

[57] Jaoko V., Taning C.N.T., Backx S., Motti P., Mulatya J., Vandenabeele J., Magomere T., Olubayo F., Mangelinckx S., Werbrouck S.P.O. (2021). Laboratory and Greenhouse Evaluation of *Melia volkensii* Extracts for Potency against African Sweet Potato Weevil, *Cylas puncticollis*, and Fall Armyworm, *Spodoptera frugiperda*. Agronomy.

[58] Melanie M., Kosasih F.Y., Kasmara H., Malini D.M., Panatarani C., Joni I.M., Husodo T., Hermawan W. (2020). Antifeedant activity of *Lantana camara* nano suspension prepared by reverse emulsion of ethyl acetate active fraction at various surfactant organic-phase ratio. Biocatal. Agric. Biotechnol..

[59] de Souza C.M., Baldin E.L.L., Ribeiro L.D.P., dos Santos T.L.B., da Silva I.F., Morando R., Vendramim J.D. (2019). Antifeedant and growth inhibitory effects of Annonaceae derivatives on *Helicoverpa armigera* (Hübner). Crop Prot..

[60] Yang H., Piao X., Zhang L., Song S., Xu Y. (2018). Ginsenosides from the stems and leaves of Panax ginseng show antifeedant activity against *Plutella xylostella* (Linnaeus). Ind. Crops Prod..

[61] Espinoza J., Urzua A., Bardehle L., Quiroz A., Echeverría J., González-Teuber M. (2018). Antifeedant Effects of Essential Oil, Extracts, and Isolated Sesquiterpenes from *Pilgerodendron uviferum* (D. Don) Florin Heartwood on Red Clover Borer *Hylastinus obscurus* (Coleoptera: Curculionidae). Molecules.

[62] Morimoto M. (2018). Insect Antifeedant Activities and Preparation of Dihydrobenzofurans from *Cyperus* spp.. ACS Symp. Ser..

[63] Baskar K., Maheswaran R., Pavunraj M., Packiam S.M., Ignacimuthu S., Duraipandiyan V., Benelli G. (2018). Toxicity and antifeedant activity of *Caesalpinia bonduc* (L.) Roxb. (Caesalpiniaceae) extracts and fractions against the cotton bollworm *Helicoverpa armigera* Hub. (Lepidoptera: Noctuidae). Physiol. Mol. Plant Pathol..

[64] Ningombam A., Ahluwalia V., Srivastava C., Walia S. (2017). Antifeedant activity and phytochemical investigation of *Millettia pachycarpa* extracts against Tobacco Leaf Eating Caterpillar, *Spodoptera litura* (Fabricius) (Lepidoptera: Noctuidae). J. Asia-Pac. Èntomol..

[65] Paul D., Choudhury M. (2016). Larvicidal and antifeedant activity of some indigenous plants of Meghalaya against 4th instar *Helicoverpa armigera* (Hübner) larvae. J. Crop Prot..

[66] Magrini F.E., Specht A., Gaio J., Girelli C.P., Migues I., Heinzen H., Saldaña J., Sartori V.C., Cesio V. (2015). Antifeedant activity and effects of fruits and seeds extracts of *Cabralea canjerana* canjerana (Vell.) Mart. (Meliaceae) on the immature stages of the fall armyworm *Spodoptera frugiperda* (JE Smith) (Lepidoptera: Noctuidae). Ind. Crops Prod..

[67] Manickam P., Kathirvelu B., Sundaram J., Munusamy A. (2014). Bio-efficacy of crude leaf extracts of *Acalypha fruticosa* Forssk. against some agriculturally important insect pests. Asian Pac. J. Trop. Dis..

[68] Lingathurai S., Vendan S.E., Paulraj M.G., Ignacimuthu S. (2011). Antifeedant and larvicidal activities of *Acalypha fruticosa* Forssk. (Euphorbiaceae) against *Plutella xylostella* L. (Lepidoptera: Yponomeutidae) larvae. J. King Saud Univ. Sci..

[69] Yadav P.A., Suresh G., Rao M.S.A., Shankaraiah G., Rani P.U., Babu K.S. (2014). Limonoids from the leaves of *Soymida febrifuga* and their insect antifeedant activities. Bioorg. Med. Chem. Lett..

[70] Sousa R.M.O.F., Rosa J.S., Oliveira L., Cunha A., Fernandes-Ferreira M. (2013). Activities of Apiaceae Essential Oils against Armyworm, *Pseudaletia unipuncta* (Lepidoptera: Noctuidae). J. Agric. Food Chem..

[71] Akhtar Y., Pages E., Stevens A., Bradbury R., da Camara C.A.G., Isman M.B. (2012). Effect of chemical complexity of essential oils on feeding deterrence in larvae of the cabbage looper. Physiol. Èntomol..

[72] Yan T.K., Asari A., Abdullah S., Ismail M., Azmi W.A. (2019). The dataset for antifeedant activity of eugenol derived compounds against red palm weevil (*Rhynchophorus ferrugineus*, Olivier) larvae. Data Brief.

[73] Hernández-Lambraño R., Caballero-Gallardo K., Olivero-Verbel J. (2014). Toxicity and antifeedant activity of essential oils from three aromatic plants grown in Colombia against *Euprosterna elaeasa* and *Acharia fusca* (Lepidoptera: Limacodidae). Asian Pac. J. Trop. Biomed..

[74] Patra J.K., Das G., Lee S., Kang S.-S., Shin H.-S. (2018). Selected commercial plants: A review of extraction and isolation of bioactive compounds and their pharmacological market value. Trends Food Sci. Technol..

[75] Nawrot J., Harmatha J. (2012). Phytochemical feeding deterrents for stored product insect pests. Phytochem. Rev..

[76] Khandelwal N., Barbole R.S., Banerjee S., Chate G.P., Biradar A.V., Khandare J.J., Giri A.P. (2016). Budding trends in integrated pest management using advanced micro- and nano-materials: Challenges and perspectives. J. Environ. Manag..

[77] Kapinder, Dangi K., Verma A.K. (2021). Efficient & eco-friendly smart nano-pesticides: Emerging prospects for agriculture. Mater. Today Proc..

[78] Suresh U., Murugan K., Panneerselvam C., Aziz A.T., Cianfaglione K., Wang L., Maggi F. (2020). Encapsulation of sea fennel (*Crithmum maritimum*) essential oil in nanoemulsion and SiO_2_ nanoparticles for treatment of the crop pest *Spodoptera litura* and the dengue vector *Aedes aegypti*. Ind. Crops Prod..

[79] Mohafrash S.M., Fallatah S.A., Farag S.M., Mossa A.-T. (2020). *Mentha spicata* essential oil nanoformulation and its larvicidal application against *Culex pipiens* and *Musca domestica*. Ind. Crops Prod..

[80] Campolo O., Giunti G., Laigle M., Michel T., Palmeri V. (2020). Essential oil-based nano-emulsions: Effect of different surfactants, sonication and plant species on physicochemical characteristics. Ind. Crops Prod..

[81] Lopes A.I.F., Monteiro M., Araújo A.R.L., Rodrigues A.R.O., Castanheira E.M.S., Pereira D.M., Olim P., Gil Fortes A., Gonçalves M.S.T. (2020). Cytotoxic Plant Extracts towards Insect Cells: Bioactivity and Nanoencapsulation Studies for Application as Biopesticides. Molecules.

[82] Rajkumar V., Gunasekaran C., Dharmaraj J., Chinnaraj P., Paul C.A., Kanithachristy I. (2020). Structural characterization of chitosan nanoparticle loaded with Piper nigrum essential oil for biological efficacy against the stored grain pest control. Pestic. Biochem. Physiol..

[83] Ahmadi Z., Saber M., Bagheri M., Mahdavinia G.R. (2018). *Achillea millefolium* essential oil and chitosan nanocapsules with enhanced activity against *Tetranychus urticae*. J. Pest Sci..

[84] Yang L., Kaziem A.E., Lin Y., Li C., Tan Y., Huang S., Cheng D., Xu H., Zhang Z. (2021). Carboxylated β-cyclodextrin anchored hollow mesoporous silica enhances insecticidal activity and reduces the toxicity of indoxacarb. Carbohydr. Polym..

[85] Pereira K.D.C., Quintela E.D., Da Silva D.J., Nascimento V.A.D., Da Rocha D.V.M., Silva J.F.A.E., Forim M.R., Silva F.G., Cazal C.D.M. (2018). Characterization of Nanospheres Containing Zanthoxylum riedelianum Fruit Essential Oil and Their Insecticidal and Deterrent Activities against *Bemisia tabaci* (Hemiptera: Aleyrodidae). Molecules.

[86] Rajkumar V., Gunasekaran C., Paul C.A., Dharmaraj J. (2020). Development of encapsulated peppermint essential oil in chitosan nanoparticles: Characterization and biological efficacy against stored-grain pest control. Pestic. Biochem. Physiol..

[87] Giunti G., Palermo D., Laudani F., Algeri G.M., Campolo O., Palmeri V. (2019). Repellence and acute toxicity of a nano-emulsion of sweet orange essential oil toward two major stored grain insect pests. Ind. Crops Prod..

[88] Oliveira J.L., Campos E.V.R., Pereira A., Nunes L.E.S., Da Silva C.C.L., Pasquoto T., Lima R., Smaniotto G., Polanczyk R.A., Fraceto L.F. (2018). Geraniol Encapsulated in Chitosan/Gum Arabic Nanoparticles: A Promising System for Pest Management in Sustainable Agriculture. J. Agric. Food Chem..

[89] Chen H., Chen L., Shen Z., Zhou H., Hao L., Xu H., Zhou X. (2020). Synthesis of mesoporous silica post-loaded by methyl eugenol as an environment-friendly slow-release bio pesticide. Sci. Rep..

[90] Attia R.G., Rizk S.A., Hussein M.A., Fattah H.M.A., Khalil M., Ma’Moun S.A. (2020). Effect of cinnamon oil encapsulated with silica nanoparticles on some biological and biochemical aspects of the rice moth, *Corcyra cephalonica* (Staint.) (Lepidoptera: Pyralidae). Ann. Agric. Sci..

[91] Paulraj M.G., Ignacimuthu S., Gandhi M.R., Shajahan A., Ganesan P., Packiam S.M., Al-Dhabi N.A. (2017). Comparative studies of tripolyphosphate and glutaraldehyde cross-linked chitosan-botanical pesticide nanoparticles and their agricultural applications. Int. J. Biol. Macromol..

[92] Zhao X., Zhu Y., Zhang C., Lei J., Ma Y., Du F. (2017). Positive charge pesticide nanoemulsions prepared by the phase inversion composition method with ionic liquids. RSC Adv..

[93] Nuruzzaman M., Liu Y., Rahman M.M., Dharmarajan R., Duan L., Uddin A.F.M.J., Naidu R. (2019). Nanobiopesticides: Composition and Preparation Methods.

[94] de Oliveira C., Mulinari J., Reichert F., Júnior A. (2020). Nano-Delivery Systems of Pesticides Active Agents for Agriculture Applications—An Overview. Ciência, Tecnologia e Inovação: Do Campo à Mesa, Proceeding of International Agribusiness Congress, Recife, Brazil, 25–27 September 2020.

[95] Kashyap P.L., Xiang X., Heiden P. (2015). Chitosan nanoparticle based delivery systems for sustainable agriculture. Int. J. Biol. Macromol..

[96] Pretty J., Bharucha Z.P. (2015). Integrated pest management for sustainable intensification of agriculture in Asia and Africa. Insects.

[97] Koul O., Cuperus G.W., Elliott N. (2008). Areawide Pest Management: Theory and Implementation.

[98] Kamaraj C., Rajakumar G., Rahuman A.A., Velayutham K., Bagavan A., Zahir A.A., Elango G. (2011). Feeding deterrent activity of synthesized silver nanoparticles using *Manilkara zapota* leaf extract against the house fly, *Musca domestica* (Diptera: Muscidae). Parasitol. Res..

[99] Siva C., Kumar M.S., Nagar G., Nadu T., Nagar G., Nadu T. (2015). Pesticidal activity of eco-friendly synthesized silver nano-particles using *Aristolochia indica* extract against *Helicoverpa armigera* Hubner (Lepidoptera: Noctuidae). Int. J. Adv. Sci. Tech. Res..

[100] Manimegalai T., Raguvaran K., Kalpana M., Maheswaran R. (2020). Green synthesis of silver nanoparticle using *Leonotis nepetifolia* and their toxicity against vector mosquitoes of *Aedes aegypti* and *Culex quinquefasciatus* and agricultural pests of *Spodoptera litura* and *Helicoverpa armigera*. Environ. Sci. Pollut. Res..

[101] González J.O.W., Gutiérrez M.M., Ferrero A.A., Band B.F. (2014). Essential oils nanoformulations for stored-product pest control—Characterization and biological properties. Chemosphere.

[102] Giongo A.M.M., Vendramim J.D., Forim M.R. (2016). Evaluation of neem-based nanoformulations as alternative to control fall armyworm. Ciência Agrotecnologia.

[103] Yeguerman C., Jesser E., Massiris M., Delrieux C., Murray A., González J.W. (2020). Insecticidal application of essential oils loaded polymeric nanoparticles to control German cockroach: Design, characterization and lethal/sublethal effects. Ecotoxicol. Environ. Saf..

[104] Zheng Q., Wang R., Qin D., Yang L., Lin S., Cheng D., Huang S., Zhang Z. (2021). Insecticidal efficacy and mechanism of nanoparticles synthesized from chitosan and carboxymethyl chitosan against *Solenopsis invicta* (Hymenoptera: Formicidae). Carbohydr. Polym..

[105] Narayanan K.B., Sakthivel N. (2010). Biological synthesis of metal nanoparticles by microbes. Adv. Colloid Interface Sci..

[106] Pascual-Villalobos M.J., Guirao P., Díaz-Baños F.G., Cantó-Tejero M., Villora G. (2019). Oil in water nanoemulsion formulations of botanical active substances. Nano-Biopesticides Today and Future Perspectives.

[107] McClements D.J., Rao J. (2011). Food-Grade Nanoemulsions: Formulation, Fabrication, Properties, Performance, Biological Fate, and Potential Toxicity. Crit. Rev. Food Sci. Nutr..

[108] Ostertag F., Weiss J., McClements D.J. (2012). Low-energy formation of edible nanoemulsions: Factors influencing droplet size produced by emulsion phase inversion. J. Colloid Interface Sci..

[109] Solans C., Solé I. (2012). Nano-emulsions: Formation by low-energy methods. Curr. Opin. Colloid Interface Sci..

[110] An C., Sun C., Li N., Huang B., Jiang J., Shen Y., Wang C., Zhao X., Cui B., Wang C. (2022). Nanomaterials and nanotechnology for the delivery of agrochemicals: Strategies towards sustainable agriculture. J. Nanobiotech..

[111] Abdollahdokht D., Gao Y., Faramarz S., Poustforoosh A., Abbasi M., Asadikaram G., Nematollahi M.H. (2022). Conventional agrochemicals towards nano-biopesticides: An overview on recent advances. Chem. Biol. Technol. Agric..

